# Characterisation and Comparison of Material Parameters of 3D-Printable Absorbing Materials

**DOI:** 10.3390/ma15041503

**Published:** 2022-02-17

**Authors:** Tobias Plüss, Felix Zimmer, Tobias Hehn, Axel Murk

**Affiliations:** 1 Institute of Applied Physics, University of Bern, 3012 Bern, Switzerland; axel.murk@unibe.ch; 2 Wehrwissenschaftliches Institut für Werk-und Betriebsstoffe (WIWeB), Institutsweg 1, 85435 Erding, Germany; felixzimmer@bundeswehr.org (F.Z.); tobiashehn@bundeswehr.org (T.H.)

**Keywords:** 3D printing, filament, material parameters, absorber materials, permittivity, permeability, material parameter measurement

## Abstract

We compared different commercially available materials that are 3D-printable for their suitability for making microwave absorbers by means of additive manufacturing, i.e., 3D printing. For this, we determined their complex permittivity, and, if applicable, the complex permeability. They are responsible for the RF losses within the material and, therefore, determine its usefulness as an absorber material. Further, we made SEM (scanning electron microscope) images of material samples showing the filling materials that have been used to achieve absorbing properties.

## 1. Introduction

Numerous applications require the use of microwave absorbers. Microwave-absorbing media are used for calibration purposes in remote sensing applications, for instance, in radiometers [1] or for radioastronomy [2]. Other microwave devices, such as mixers, circulators, or directional couplers, also use microwave absorbers in the form of terminations [3], and even entire microwave devices can be 3D printed [4]. Microwave absorbers can be used to eliminate or reduce stray radiation, so-called radio frequency interference (RFI), that could interfere with other systems [5], or to suppress unwanted oscillations caused by cavity resonances. There are many different material types that can be used as absorbing media [6], for example, epoxy with filler materials, such as Eccosorb [7] or Steelcast [8]. Silicone with filler materials is also used, as well as thermoplasts such as polypropylene [9]. However, these materials have several drawbacks: when the epoxy resin-based materials are used, one needs appropriate casting moulds and the absorber needs to be cast and cured before it can be used. For the thermoplasts, injection moulding is used for manufacturing, but again, this requires a special mould. The need for a special mould makes these techniques and materials expensive and time-consuming, due to the mould manufacturing and/or curing cycles required even for small quantities and rapid prototyping. Especially for such use cases, 3D printing is an interesting technique that allows many shapes to be produced quickly and at low cost [10,11,12] because no casting moulds are needed, no curing cycle needs to be followed, and no resin needs to be prepared. In a more general sense, 3D printing is not only attractive for absorbers, but for producing microwave and even millimeter and submillimeter wave components, such as waveguides, horn antennas, dielectric lenses, and so on [13].

Therefore, in this work, we concentrate our study on materials that are 3D-printable, namely, with the FFF method (fused filament fabrication), where the raw material has the form of a filament that is extruded. We focus on commercially available materials, most of which are readily available.

For the design, simulation, and optimisation of such microwave devices, the complex magnetic permeability $\hat{\mu }$ and the complex dielectric permittivity $\hat{\epsilon }$ need to be known.

In this report, we present different materials which we have measured, their material properties, and SEM images of material samples that show the constituents. Due to the 3D-printing process, we expect anisotropic effects in the material parameters; we show two examples of anisotropic materials and present how we measured the anisotropic effects.

## 2. Materials and Methods

### 2.1. Materials Used

We characterised several different materials that are suitable for 3D printing, based either on PLA (polylactic acid) or on ABS (acrylonitrile butadiene styrene). For the material from SAAB, the chemical composition and other ingredients are not known to us. Table 1 shows the materials we have used for this study. Throughout this work, we use the shortcut names shown in the “abbreviation” column to identify the different materials. The materials consist of a matrix and a filler which, as we will show later, has the form of a powder whose grains are embedded in the matrix. The weight percentage of the filler can be found from the material safety data sheets (MSDS) from the manufacturer [14]. Note that the MSDS is not publicly available for all materials.

### 2.2. Sample Preparation

In order to characterise the material properties in terms of magnetic permeability and dielectric permittivity, we used different measurement setups as follows:

- a rectangular waveguide resonator;
- a coaxial transmission line;
- a piece of rectangular waveguide.

Each measurement setup requires samples to be prepared in a certain shape. We used similar coaxial and waveguide measurement setups to those shown in [15,16]. The resonator method has also been used by others with success [17]. The same basic principles can also be used with different kinds of resonators [16].

The following sections describe these measurement setups, show how the samples were prepared, and explain how material parameters were extracted from the measurement data.

**Figure 1:**
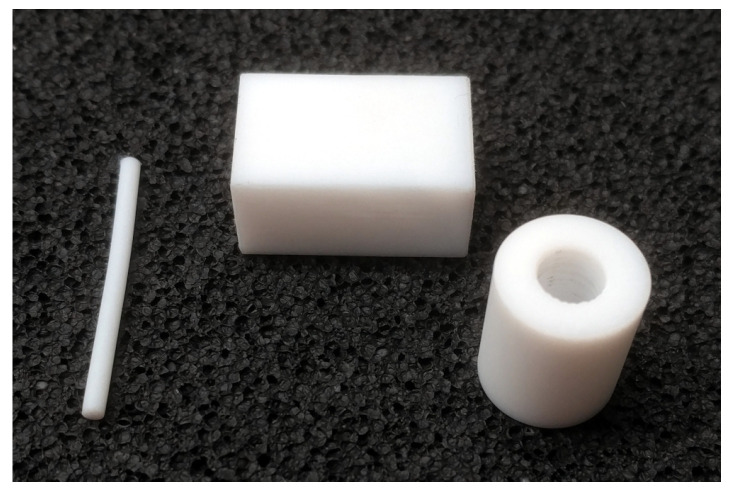
PTFE Material samples for the different measurement techniques: 2-mm diameter rod for the resonator, brick for the waveguide, and annular cylinder for the coaxial transmission line.

For verification purposes, we used pieces of PTFE since its material properties are well known: ${\hat{\mu }}_{r}=1$, ${\hat{\epsilon }}_{r}=2.08-0.0005\;i$ [18,19,20]. Figure 1 shows a photograph of the PTFE samples for our three different measurement methods. Further, for comparison, we have a printed sample of pure PLA such that the effects of the additives on the permittivity and/or permeability can be understood better in the three Protopasta PLA-based materials considered.

#### 2.2.1. Resonator Samples

For the resonator measurements, we prepared samples in the form of long, thin cylinders, which were produced by extruding a piece of filament through the nozzle of a 3D printer without actually printing. Samples with diameters between 0.4 mm and 0.8 mm were produced in this way.

#### 2.2.2. Coaxial Samples

**Figure 2:**
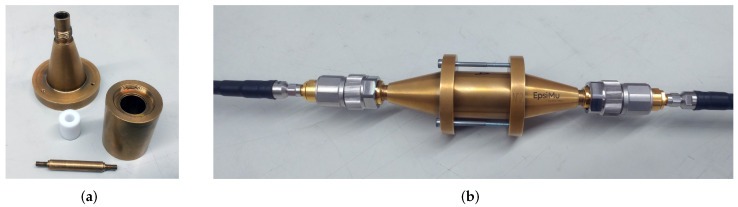
Coaxial transmission line, MUT, and connection with the network analyser. (**a**) Coaxial taper, sample holder (outer conductor) with PTFE material sample, and inner conductor; (**b**) assembled device with adaptors connected to the network analyser.

For measurements from 10 MHz to 8 GHz, we used a coaxial transmission line “EpsiMu” from Multiwave [21], as shown in Figure 2. This device consists of a sample holder which forms the outer conductor of the coaxial transmission line, an inner conductor, and adaptors to connect the 2.92-mm connectors from the network analyser. MUT (material under test) samples were prepared in the form of an annular ring which has an outer diameter of 13 mm and an inner (hole) diameter of 5.6 mm. The samples were 3D-printed slightly oversized and then machined to fit precisely into the sample holder. With the samples in place, the adaptors were assembled and the S-parameters measured with a Rohde & Schwarz ZVA40 vector network analyser. If the sample holder is air-filled, the geometry of this transmission line results in a 50-Ω impedance. Due to the dimensions of this coaxial transmission line, higher-order modes can propagate above 10 GHz, which would affect the measurement; therefore, we use the coaxial transmission line only up to 8 GHz. Prior to the measurements, the network analyser was calibrated with an automatic calibration unit and the port extension feature was used to compensate for the additional connectors and adaptors used.

#### 2.2.3. Waveguide Samples

**Figure 3:**
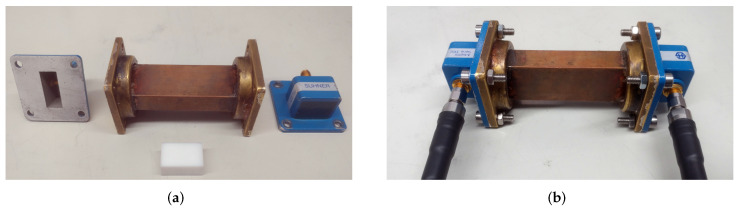
Waveguide as sample holder, MUT, and connection with the network analyser. (**a**) WR90 waveguide with coaxial adaptors and PTFE material sample; (**b**) assembled waveguide connected to the network analyser.

For the frequency range from 7 GHz to 13 GHz, we used a piece of WR-90 (X-band) waveguide and a set of coaxial-to-waveguide adaptors, as shown in Figure 3. The MUT samples were prepared in the form of small bricks having dimensions slightly larger than the waveguide. After 3D-printing the samples, they were machined to fit precisely into the waveguide. With the samples in place, the coaxial to waveguide adaptors were assembled and the S-parameters were measured. Prior to the S-parameter measurement, the network analyser was calibrated using the TRM (thru-reflect-match) method: for the “thru” standard, we connected the coxial to waveguide adaptors back-to-back; for the “reflect” standard, we used a shorting plate directly in front of the coaxial to waveguide adaptors, and for the “match” standard, a waveguide load was used.

#### 2.2.4. Samples for SEM Imaging

For the SEM (scanning electron microscope) images, we used one small block of material that was 3D-printed from CDP material to see what the extruded material looked like. To investigate the constituents of the materials, we used pieces of unprocessed filament. The filament was broken apart to create a fresh fracture surface that was then investigated using a SEM.

### 2.3. Material Parameter Extraction

To extract the material parameters, namely, the complex permittivity, ${\hat{\epsilon }}_{r}=\epsilon_{r}^{\prime }+i\;\epsilon_{r}^{\prime \prime }$, and, where applicable, the complex permeability, ${\hat{\mu }}_{r}=\mu_{r}^{\prime }+i\;\mu_{r}^{\prime \prime }$, we employed different methods that are all based on the transmission and/or reflection S-parameters $S_{11}$, $S_{21}$, $S_{12}$, and $S_{22}$. The following sections describe how we extracted the material parameters using different techniques.

#### 2.3.1. Resonator Method

**Figure 4:**
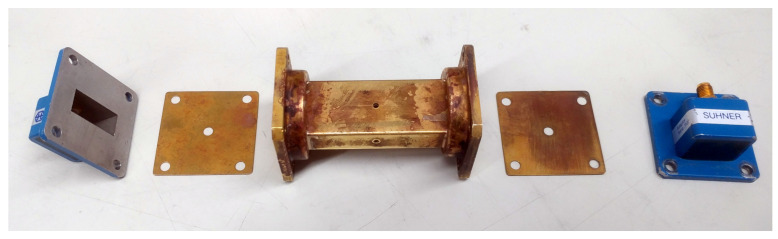
WR90 waveguide with coupling irises and coaxial to waveguide adaptors. The small holes in the waveguide’s broad and narrow walls are used for sample insertion.

The resonator method uses a piece of WR90 waveguide that has small holes in its broad and narrow walls where MUT samples can be inserted. A resonator is constructed by using two coupling irises on both ends of the waveguide, as shown in Figure 4.

The resonant frequency of this rectangular cavity resonator is given, in general, by [18,22]:

(1) $$f_{mnp}=\frac{c}{2}\sqrt{\frac{m^{2}}{a^{2}}+\frac{n^{2}}{b^{2}}+\frac{p^{2}}{\ell^{2}}},$$

with *c* being the speed of light, *a* and *b* being the broad and narrow wall dimensions of the waveguide, respectively, and *ℓ* being the length between the two irises; *m*, *n*, and *p* are the mode numbers. We restricted ourselves to TE${}_{10p}$ modes such that m=1 and n=0; therefore,

(2) $$f_{p}=\frac{c}{2}\sqrt{\frac{1}{a^{2}}+\frac{p^{2}}{\ell^{2}}}$$

is the resulting resonant frequency for the *p*-th mode. To extract the material parameters, the cavity perturbation method is employed as described in [23,24]. We measured the transmission coefficient $S_{21}$ of the empty resonator with the network analyser and determined the resonant frequency and *Q* from the resulting frequency response. Then, a material sample was inserted through the holes and the measurement was repeated. The complex permittivity can be determined with:

(3) $$\epsilon_{r}^{\prime }=\frac{f_{0}-f_{m}}{2\;f_{m}}\frac{V_{0}}{V_{m}}+1,$$

and:

(4) $$\epsilon_{r}^{\prime \prime }=\frac{V_{0}}{4\;V_{m}}\left(\frac{1}{Q_{0}}-\frac{1}{Q_{m}}\right),$$

where $V_{0}$ and $V_{m}$ are the volume of the resonator and the sample, $f_{0}$ and $f_{m}$ are the resonant frequencies with and without a sample, and $Q_{0}$ and $Q_{m}$ are the resonator’s *Q* with and without a sample. The complex permeability can be determined from:

(5) $$\mu_{r}^{\prime }=\frac{\ell^{2}+a^{2}\;p^{2}}{2\;a^{2}\;p^{2}}\frac{f_{0}-f_{m}}{f_{m}}\frac{V_{0}}{V_{m}}+1,$$

and:

(6) $$\mu_{r}^{\prime \prime }=\frac{\ell^{2}+a^{2}\;p^{2}}{4\;a^{2}\;p^{2}}\frac{V_{0}}{V_{m}}\left(\frac{1}{Q_{0}}-\frac{1}{Q_{m}}\right)\;.$$

**Figure 5:**
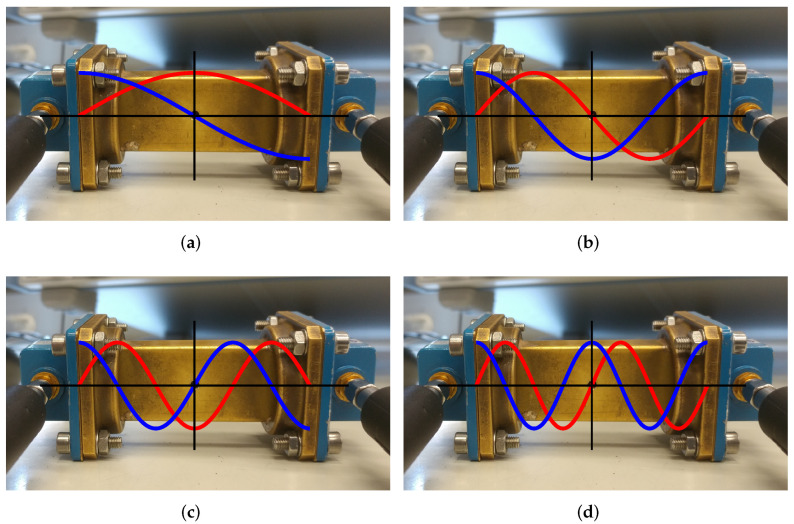
Waveguide rectangular resonator with schematic representation of the electric (red lines) and magnetic (blue lines) field distributions for different modes. (**a**) TE${}_{101}$ mode, p=1; (**b**) TE${}_{102}$ mode, p=2; (**c**) TE${}_{103}$ mode, p=3; (**d**) TE${}_{104}$ mode, p=4.

Note that, for modes having an odd number *p*, the electric field in the centre of the resonator has a maximum and the magnetic field is null. On the other hand, for even numbers *p*, the electric field in the centre of the resonator is null and the magnetic field has a maximum. Figure 5 schematically illustrates the amplitudes of the electric and magnetic fields for different modes, with the red lines indicating the electric field and the blue lines indicating the magnetic field.

**Figure 6:**
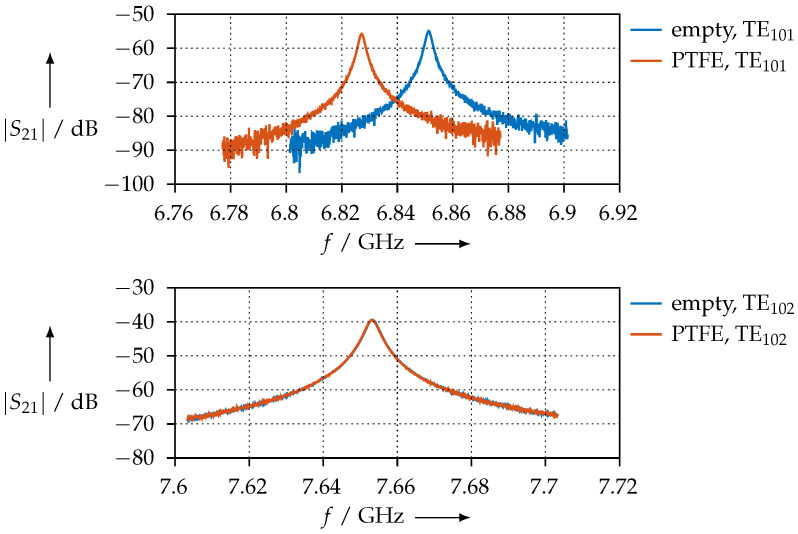
Example of the frequency response of the rectangular waveguide resonator with and without PTFE material sample for TE${}_{101}$ (**upper** chart) and TE${}_{102}$ (**lower** chart) modes.

Therefore, for the “odd” modes, only the permittivity can be determined (because the magnetic field is null where the sample is placed), whereas for “even” modes, only the permeability can be determined (because the electric field is null where the sample is placed). Note that the sample is always placed such that it is parallel to the field, i.e., for the permittivity measurement, the sample is perpendicular to the broad wall, and for the permeability measurement, the sample is perpendicular to the narrow wall. As an example, Figure 6 shows the measured $S_{21}$ frequency responses for this resonator when it is empty or when a PTFE sample is inserted. The TE${}_{101}$ mode is affected due to the dielectric properties of PTFE, i.e., its resonant frequency and *Q* are slightly lower when the PTFE sample is inserted. On the other hand, the TE${}_{102}$ mode is unaffected since PTFE has no magnetic properties (${\hat{\mu }}_{r}=1$).

To determine the resonant frequency and *Q* of the resonator, we used the Nelder–Mead method to fit a Lorentzian function,

(7) $${\hat{S}}_{21}=\frac{A}{1+i\;Q\left(\frac{f}{f_{p}}-\frac{f_{p}}{f}\right)},$$

to the measured $S_{21}$ data with the fit parameters *A*, *Q*, and $f_{p}$. This gives better results than if only the 3-dB bandwidth and the frequency at the $S_{21}$ peak were to be measured, as these measurements rely only on three data points, whereas our approach takes into account the entire frequency response around the resonance [25].

Table 2 shows the resonant frequency and *Q* values that have been measured for the PTFE reference sample together with the permittivity and permeability values calculated. As we can see, the permittivity is very close to the value that is reported in the literature [18,19,20], and the permeability is, as expected, very close to 1. This demonstrates the suitability of our rectangular waveguide cavity resonator for material parameter extraction.

However, the resonator method has one obvious drawback: it is not possible to simply obtain the material parameters for a broad frequency range, i.e., one can obtain the material parameters only for a set of discrete frequencies.

#### 2.3.2. Transmission/Reflection Method

**Figure 7:**
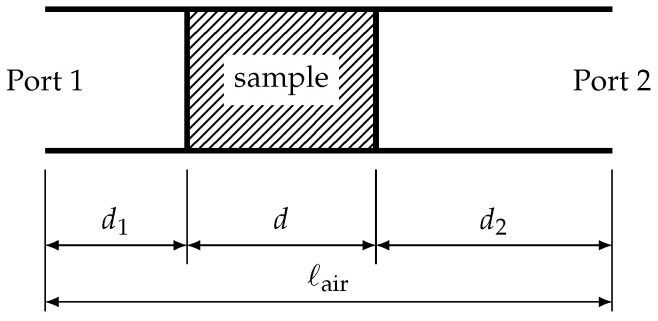
Schematic representation of the measurement setup in a waveguide.

For the transmission/reflection method, we used either the coaxial cell or a piece of waveguide where the MUT sample was inserted. A schematic representation of the measurement setup is shown in Figure 7. The measurement setup, be it coaxial or waveguide, has two ports, at which all four S-parameters are measured. In contrast to the resonator method, the transmission/reflection method allows for the determination of the material parameters for a broad frequency range.

From the S-parameters, we extracted the material parameters using Matlab by means of the “Baker–Jarvis Reference Plane Invariant Method” [26]. This method works in the same way for both coaxial and waveguide measurements. In brief, the extraction process works as follows. Let

(8) $$\gamma_{0}=i\sqrt{{\left(\frac{\omega }{c}\right)}^{2}-{\left(\frac{2\;\pi }{\lambda_{c}}\right)}^{2}}$$

be the propagation constant in a coaxial transmission line or in a waveguide that is air-filled; $\lambda_{c}$ denotes the cutoff wavelength, which is +∞ for a coaxial transmission line and 2a for a rectangular waveguide when *a* is the length of the waveguide broad wall [22]. Then,

(9) $$\gamma =i\sqrt{{\left(\frac{\omega }{c}\right)}^{2}\;{\hat{\epsilon }}_{r}\;{\hat{\mu }}_{r}-{\left(\frac{2\;\pi }{\lambda_{c}}\right)}^{2}}$$

is the propagation constant within the MUT sample. The transmission coefficient *inside* the sample is:

(10) $$T=e^{-\gamma \;d},$$

with *d* being the sample thickness (refer to Figure 7); the reflection coefficient *at the sample faces* can be expressed as:

(11) $$R=\frac{\frac{\gamma_{0}}{\mu_{0}}-\frac{\gamma }{\mu_{0}\;{\hat{\mu }}_{r}}}{\frac{\gamma_{0}}{\mu_{0}}+\frac{\gamma }{\mu_{0}\;{\hat{\mu }}_{r}}}=\frac{\gamma_{0}-\frac{\gamma }{{\hat{\mu }}_{r}}}{\gamma_{0}+\frac{\gamma }{{\hat{\mu }}_{r}}}=\frac{\gamma_{0}\;{\hat{\mu }}_{r}-\gamma }{\gamma_{0}\;{\hat{\mu }}_{r}+\gamma }\;.$$

Then, with the offsets $d_{1}$ and $d_{2}$ (refer to Figure 7), the reflection coefficient at port 1 can be expressed as:

(12) $$S_{11}=\frac{\Gamma \left(1-z^{2}\right)}{1-\Gamma^{2}\;z^{2}}\cdot e^{-\gamma_{0}\;2\;d_{1}},$$

and, similarly:

(13) $$S_{22}=\frac{\Gamma \left(1-z^{2}\right)}{1-\Gamma^{2}\;z^{2}}\cdot e^{-\gamma_{0}\;2\;d_{2}}\;.$$

The transmission coefficients in both the forward and backward direction are expressed as follows:

(14) $$S_{21}=S_{12}=\frac{z\left(1-\Gamma^{2}\right)}{1-\Gamma^{2}\;z^{2}}\cdot e^{-\gamma_{0}\;\left(d_{1}+d_{2}\right)}.$$

Note that $d_{1}+d_{2}$ in the exponent is the same as $\ell_{\mathrm{air}}-d$. If we use the following combinations of S-parameters, namely,

(15) $$S_{21}\;S_{12}-S_{11}\;S_{22}=e^{-2\;\gamma_{0}\left(\ell_{\mathrm{air}}-d\right)}\cdot \frac{T^{2}-R^{2}}{1-T^{2}\;R^{2}}$$

and

(16) $$\frac{1}{2}\left(S_{21}+S_{12}\right)=e^{-\gamma_{0}\left(\ell_{\mathrm{air}}-d\right)}\cdot \frac{T\left(1-R^{2}\right)}{1-T^{2}\;R^{2}},$$

then we can see that the air gaps $d_{1}$ and $d_{2}$ are effectively eliminated, and only knowledge about the length of the sample holder, $\ell_{\mathrm{air}}$, and of the sample itself, *d*, is required.

Equations (15) and (16) are complex equations; if expressed as real and imaginary parts or as magnitude and phase, these are, in fact, 4 real equations for 2 unknowns if we are interested in either the permittivity or the permeability only, and for 4 unknowns if we are interested in both. For our material parameter-extraction method, we simultaneously solve the real and imaginary parts of Equations (15) and (16) by means of the Nelder–Mead method using Matlab. The calculation steps are exactly the same, no matter whether the measurement setup is a coaxial transmission line or a waveguide since the cutoff wavelength is taken into account in Equations (8) and (9). The drawback of using the Nelder–Mead method for the material parameter extraction is that this method requires an initial guess for the material parameters to be extracted. To determine the initial guess, we can use the resonator method.

**Figure 8:**
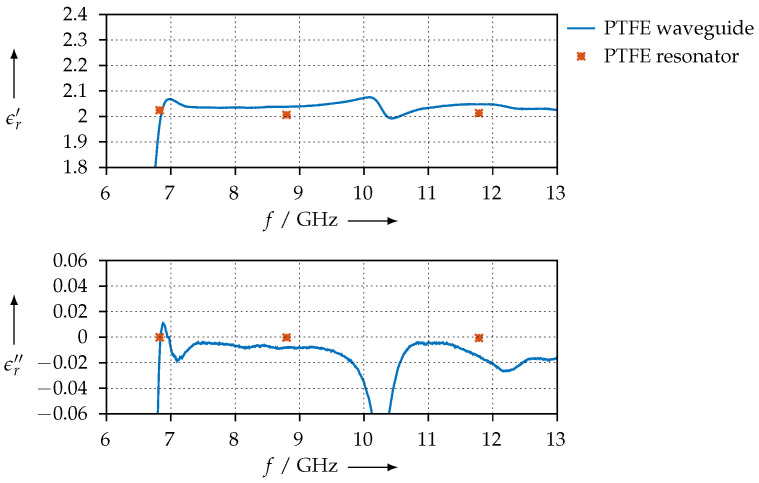
Extracted permittivity of the PTFE reference sample and comparison with the values that have been measured with the resonator method.

For reference, Figure 8 shows the permittivity that we extracted for the PTFE sample using the waveguide method and compares the values with the ones that have been obtained with the resonator. Except for the low end at 6.6 GHz, which is close to the waveguide’s cutoff frequency, we observe good agreement between the two methods.

**Figure 9:**
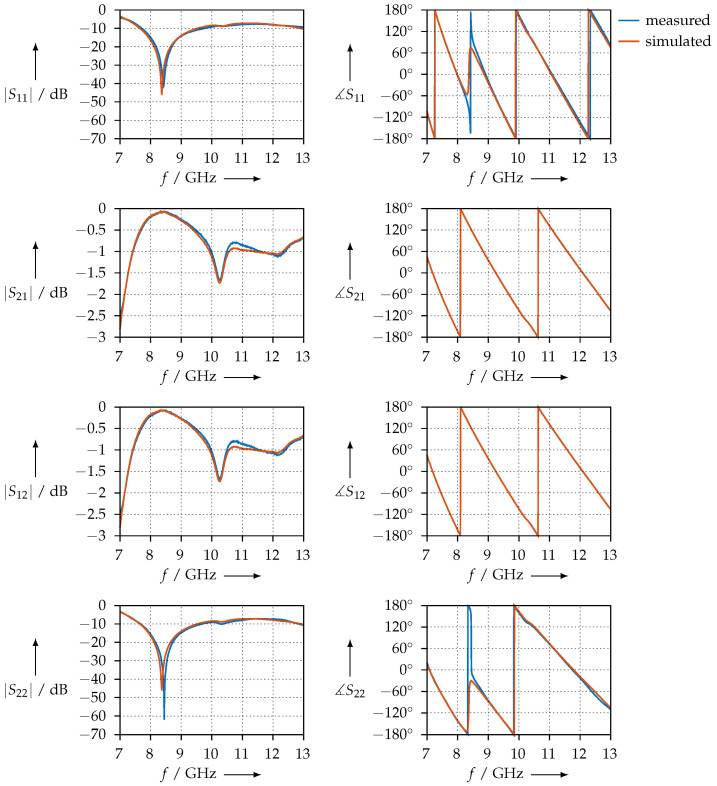
Measured and simulated S-parameters of the PTFE sample in the waveguide.

Figure 9 shows the measured S-parameters for the PTFE sample. To assess the measurement, the following conditions need to be observed:

- the magnitude of $S_{11}$ and $S_{22}$ should be equal, i.e., the sample should have the same reflection coefficient, no matter the direction from which the waves travel;
- the magnitude and phase of $S_{21}$ and $S_{12}$ should be equal, i.e., the transmission through the samples does not depend on the direction.

As we can observe from Figure 9, the two conditions are satisfied and the measurement is consistent. We also plotted the simulated S-parameters: using the permittivity shown in Figure 8, we calculated $S_{11}$, $S_{21}$, $S_{12}$, and $S_{22}$ with the aid of Equations (12)–(14) and plotted the S-parameters calculated in this way together with the measured ones.

**Figure 10:**
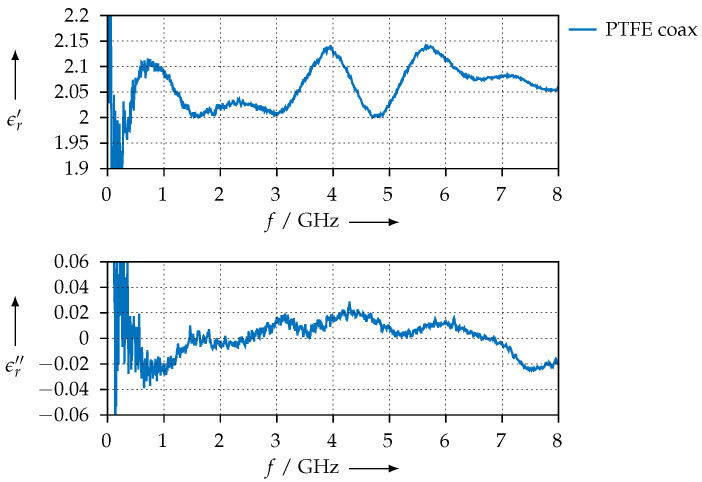
Extracted permittivity of the PTFE reference sample measured with the coaxial setup.

**Figure 11:**
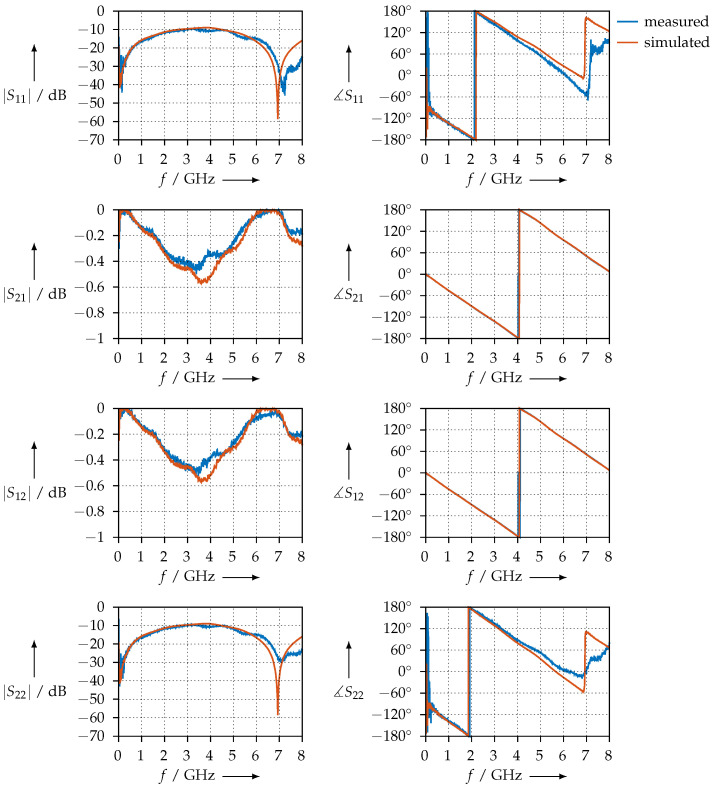
Measured and simulated S-parameters of the PTFE sample in the coaxial transmission line.

For comparison, Figure 10 shows the permittivity that was measured for the PTFE sample using the coaxial transmission line. The S-parameters, both measured and simulated, are shown in Figure 11.

### 2.4. Considerations in the Context of Anisotropic Material Parameters

**Figure 12:**
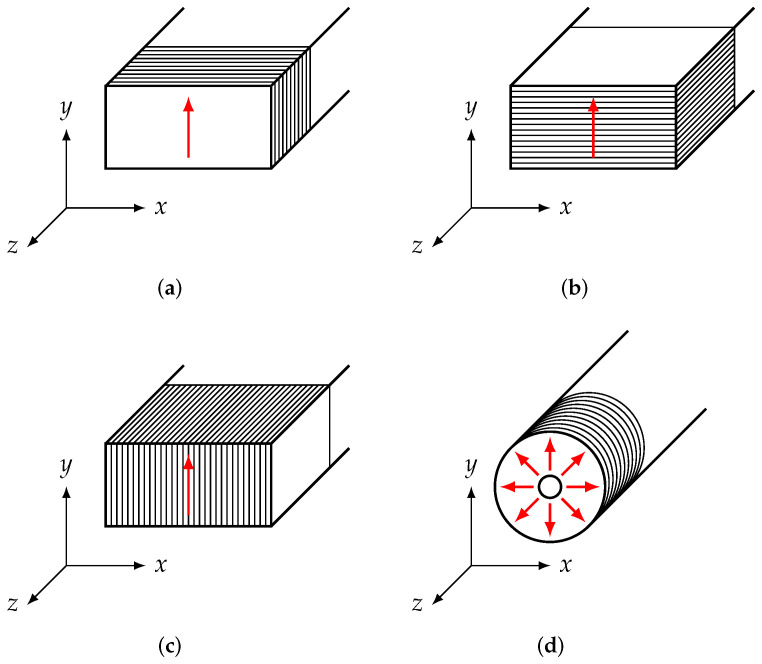
Waveguide and coaxial samples with electric field orientation indicated. (**a**) Waveguide, xy orientation; (**b**) waveguide, xz orientation; (**c**) waveguide, yz orientation; (**d**) coaxial.

As we will show in our results, the process of 3D printing leads us to expect that the material parameters should be anisotropic to some degree, i.e., the material parameters will depend on the way in which the MUT sample was printed. To illustrate this, consider the geometries shown in Figure 12. The 3D printer will produce the MUT samples layer by layer. These layers can be oriented in different ways that are shown in Figure 12. The waveguide’s broad wall is aligned with the *x*-axis, whereas the short wall is aligned with the *y*-axis. The electromagnetic waves travel in the ±z-direction.

In a waveguide, when the fundamental Mode TE${}_{10}$ mode is used, the electric field has a linear polarisation in the *y*-direction, which is indicated by the red arrow in Figure 12. Therefore, it is possible to produce 3 different MUT samples, where the electric field is either parallel or perpendicular to the layers printed.

In contrast to that, the coaxial transmission line uses a pure TEM wave, as shown in Figure 12, and therefore, the electric field is always parallel to the layers printed.

### 2.5. Overview of Samples Prepared

We have prepared a number of samples for each material that we have listed in Table 1. For this, we used an Ultimaker-2 printer. It should also be noted that we intentionally prepared samples of different lengths to verify the repeatability of the measurements. An overview of all MUT samples is given in Table 3. Since some materials are more complicated to process with good results, we did not produce all possible variants from all materials. In particular, CDP and KIM were especially easy to process with good repeatability; therefore, we chose these two materials to produce samples for each possible orientation to investigate the anisotropic properties.

## 3. Results

### 3.1. Microscope Images

We have examined samples of CDP, FEP, SSP, and KIM under a scanning electron microscope. This section presents our findings.

**Figure 13:**
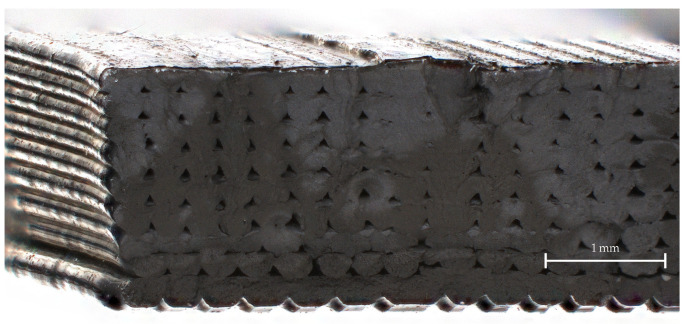
Microscope image of a printed CDP sample, low magnification.

**Figure 14:**
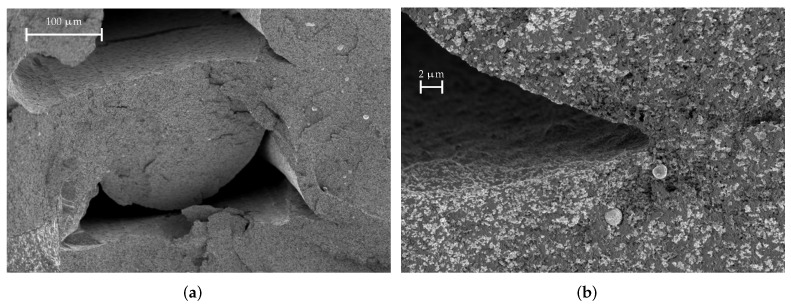
SEM images of a printed CDP material sample. (**a**) CDP, medium magnification; (**b**) CDP, large magnification.

Figure 13 shows a microscope image of a printed CDP material sample that was taken with a confocal laser scanning microscope. We can clearly observe the layered structure of the sample and the parallel-aligned, straight strands that originate from the extrusion from the 3D printer. Because the 3D-printed samples all have this structure, we expect that the material samples should exhibit anisotropic properties to some degree. For example, if the electric field is oriented parallel to the strands, we expect the permittivity to be slightly different than if the field was perpendicular to them. Moreover, the air pockets between the strands will further affect the permittivity. The same sample is shown with a much larger magnification in the SEM images of Figure 14.

**Figure 15:**
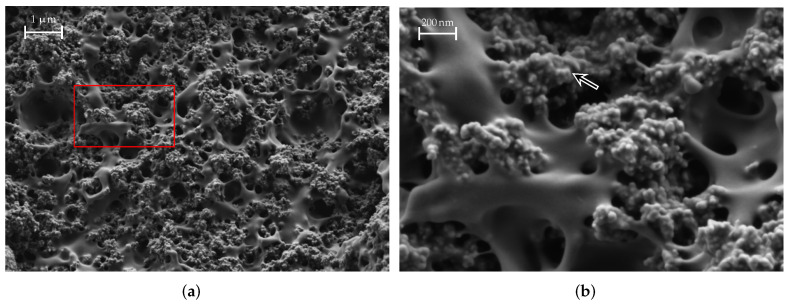
SEM images of a CDP filament material sample. The red-marked area is shown enlarged on the right. The arrow marks a chunk of filler material, which is carbon black. The smooth structure is the PLA matrix. (**a**) CDP, medium magnification; (**b**) CDP, large magnification.

A SEM image of a fresh fracture surface of a CDP filament is shown in Figure 15. The area marked with the red rectangle is shown enlarged on the right. The smooth structure that can be seen is the PLA matrix in which the filler material, carbon black in this case, is embedded. The cauliflower-like structures, one of which is marked by the arrow in Figure 15b, are carbon black conglomerates. As we can observe from the SEM images, this material as a high volume filling factor: there are so many carbon particles that they touch each other and, thus, form “bridges” that lead to a DC (direct current) conductivity of the material, which is also indicated in the material data sheet from the manufacturer. In contrast, the other materials exhibit a lower volume filling factor and, therefore, the particles are isolated within the matrix and no DC conductivity is present.

**Figure 16:**
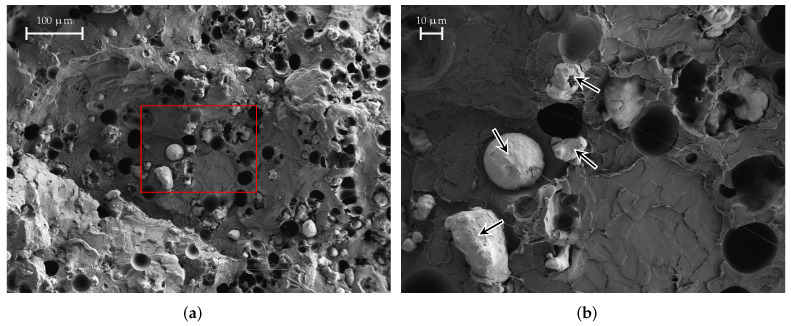
SEM images of a FEP material sample. The arrows indicate iron grains. The area marked in red is shown enlarged on the right. (**a**) FEP, medium magnification; (**b**) FEP, large magnification.

Figure 16 shows a SEM image of a FEP filament material sample. Again, the area marked in red is shown enlarged in Figure 16b. The FEP material is PLA, which is loaded with iron powder; the PLA matrix can be clearly visible, some of which are highlighted by arrows in Figure 16b. The dark, almost black round structures are small holes that originate from iron powder grains that were ripped off when the sample was prepared. Apparently, the grains are around 5 μm to 30 μm in size. As mentioned before, the individual grains are isolated in the matrix and do not touch each other; therefore, the material does not have a DC conductivity and its losses are lower.

**Figure 17:**
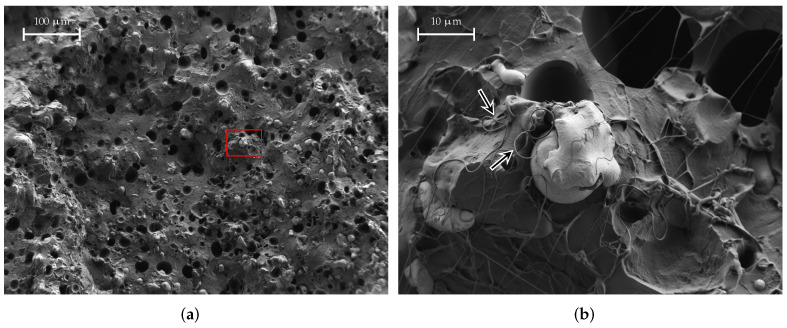
SEM images of a SSP material sample. The hair-like structures marked are actually PLA from breaking the filament sample apart. The red-marked area is shown enlarged on the right. (**a**) SSP, medium magnification; (**b**) SSP, large magnification.

A SEM image of a SSP material sample is shown in Figure 17. We can again identify the small holes that were mentioned before. Furthermore, the stainless-steel grains are also clearly visible. Further, in Figure 17b, we have marked some hair-like structures with arrows. These hair-like structures originate from the PLA matrix when the sample is prepared. We observe a grain size between approx. 5 μm and 20 μm.

**Figure 18:**
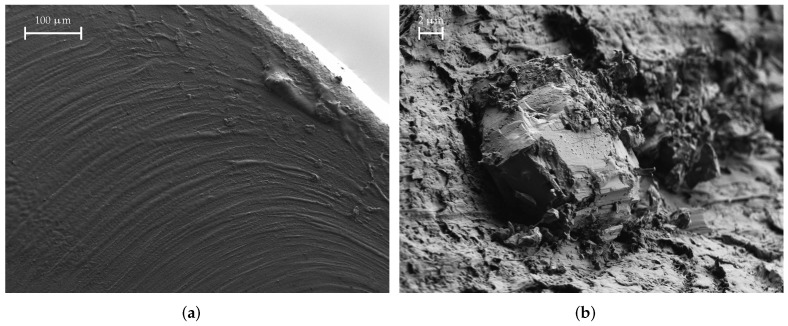
SEM images of a KIM material sample. The fracture surface is very clean and smooth and no filler grains can be identified; however, a couple of irregularities in the form of inclusions can be found. (**a**) KIM, medium magnification; (**b**) KIM, large magnification.

The fracture surfaces of the KIM material are especially interesting. Figure 18 shows a SEM image thereof. In contrast to all other materials we have seen so far, the fracture surfaces are extremely smooth. Otherwise, we could not identify any filler materials. Further, there are only very few inclusions, the material or origin of which we cannot identify; one example is shown in Figure 18b.

### 3.2. Resonator Measurements

Table 4 shows the real and imaginary parts of the permittivity and permeability we measured for all samples. Note that we performed the measurement of the empty resonator before each actual MUT measurement. For better clarity, we omitted these “empty” measurements in Table 4, showing only the MUT measurements.

We conclude the following from these measurements:

- For the PLA sample, the permittivity we found is close to the literature values; see Section 3.3.1;
- As the CDP material is PLA with carbon loading, we expect no magnetic effects. Indeed, $\mu_{r}^{\prime }$ is close to 1 and $\mu_{r}^{\prime \prime }$ is close to 0. Therefore, we can indeed conclude that no magnetic effects are present in this material; the fact that our ${\hat{\mu }}_{r}$ is different from 1 is likely due to measurement errors. On the other hand, we have two measurements of $\epsilon_{r}^{\prime }$ and $\epsilon_{r}^{\prime \prime }$, both of which are quite close together. When $\epsilon_{r}^{\prime \prime }$ of CDP is compared to the other materials, we see that the losses of the CDP material are much larger than for all other materials. The losses are so high that we were unable to reliably measure a resonance for the TE${}_{101}$ mode. Additionally, the difference between the TE${}_{103}$ and TE${}_{105}$ is larger when compared to the other materials; the reason for this is the increase in the numeric errors when the $f_{m}$ and $Q_{m}$ are determined from weak resonances;
- For the FEP material, we see that the permittivity measurements all agree very well with each other. Since FEP is a PLA matrix which is loaded with iron powder, magnetic effects could be expected to some degree; we see that there is a certain amount of magnetic loss, as $\mu_{r}^{\prime \prime }$ is clearly greater than zero. Furthermore, we also see that $\mu_{r}^{\prime }$ is slightly smaller than 1, i.e., the material is only slightly magnetic. The order of magnitude for our $\mu_{r}^{\prime }$ and $\mu_{r}^{\prime \prime }$ is comparable to the one that others report for similar materials [27];
- The SSP material is PLA loaded with stainless steel powder. We can see that $\mu_{r}^{\prime }$ is closer to 1 than for the FEP, i.e., magnetic effects are weaker for the SSP material, which makes sense, as stainless steel is generally nonmagnetic or only weakly magnetic compared to iron. Furthermore we also see that the magnetic losses are smaller than for FEP, which is again consistent with the fact that stainless steel has fewer magnetic properties than iron;
- For the KIM material, we see that all permittivity measurements agree well with each other. The permeability is close to 1, from which we conclude that this material is nonmagnetic.

### 3.3. Permittivity

#### 3.3.1. Pure PLA

**Figure 19:**
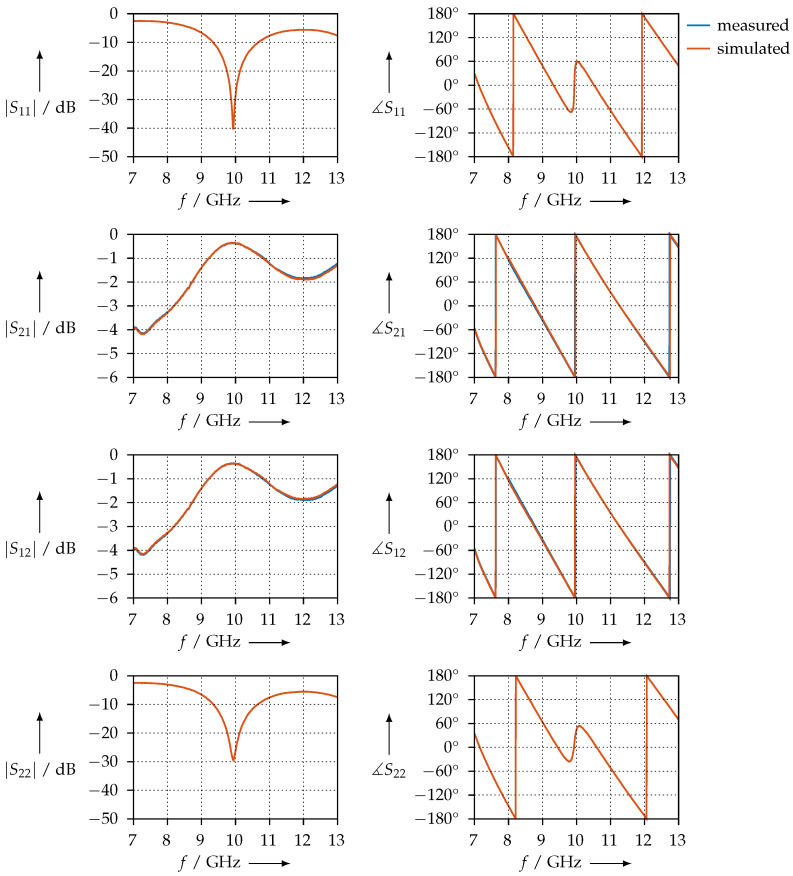
Measured and simulated S-parameters of the PLA sample in the waveguide.

The pure PLA sample is measured so that the effect of the filler materials can be identified. As an example, for the 15-mm sample, the measured and simulated S-parameters are shown in Figure 19. Like for the PTFE sample (Figure 9), we observe that the magnitudes $\left|S_{11}\right|\;=\;\left|S_{22}\right|$ agree very well up to only small differences. Otherwise, the condition $S_{12}=S_{21}$ is also satisfied, i.e., the measurement is consistent and we can conclude that the permittivity extraction will yield sensible data.

**Figure 20:**
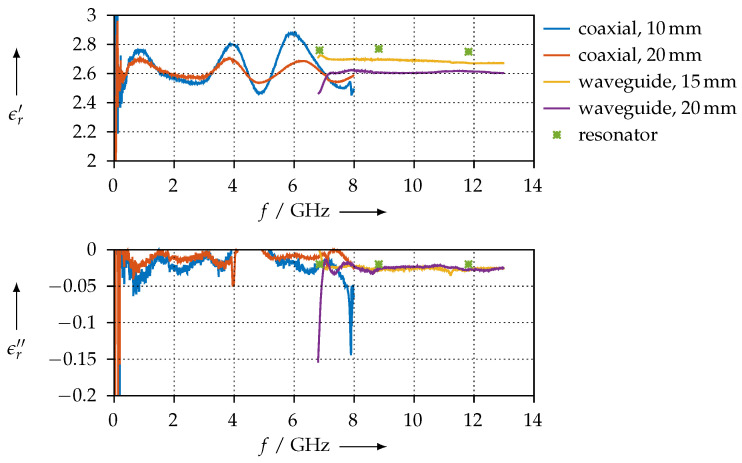
Real ($\epsilon_{r}^{\prime }$) and imaginary ($\epsilon_{r}^{\prime \prime }$) parts of the permittivity for the PLA material.

The permittivity that we calculated for the PLA sample is shown in Figure 20 for both the coaxial and the waveguide measurements of all different length samples that we made. The permittivity value we found is in good accordance with other publications [28,29,30]. Moreover, we have good agreement between the resonator and the transmission/reflection measurements.

#### 3.3.2. FEP Material

Since the FEP material consists of PLA that is loaded with iron powder, the question arises whether it exhibits magnetic properties at our test frequencies. Our extraction technique can be used in two different ways:

1. We can assume ${\hat{\mu }}_{r}=1$ and calculate ${\hat{\epsilon }}_{r}$;
2. We can calculate both ${\hat{\mu }}_{r}$ and ${\hat{\epsilon }}_{r}$ at the same time.

**Figure 21:**
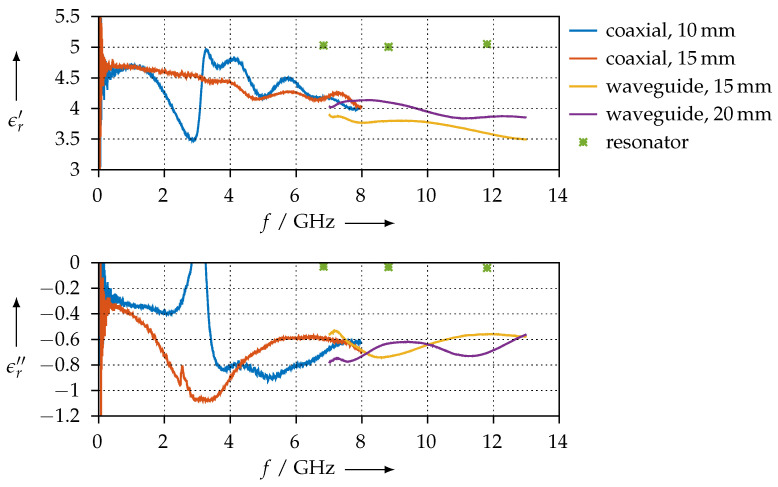
Real ($\epsilon_{r}^{\prime }$) and imaginary ($\epsilon_{r}^{\prime \prime }$) parts of the permittivity for the FEP material.

Figure 21 shows the resulting permittivity when we assume ${\hat{\mu }}_{r}=1$. As one can see, the coaxial and waveguide measurements agree very well (between 7 GHz and 8 GHz). However, we can observe a large ripple for the 10-mm coaxial sample that is most likely related to the network analyser calibration, where we have used the port extension feature.

**Figure 22:**
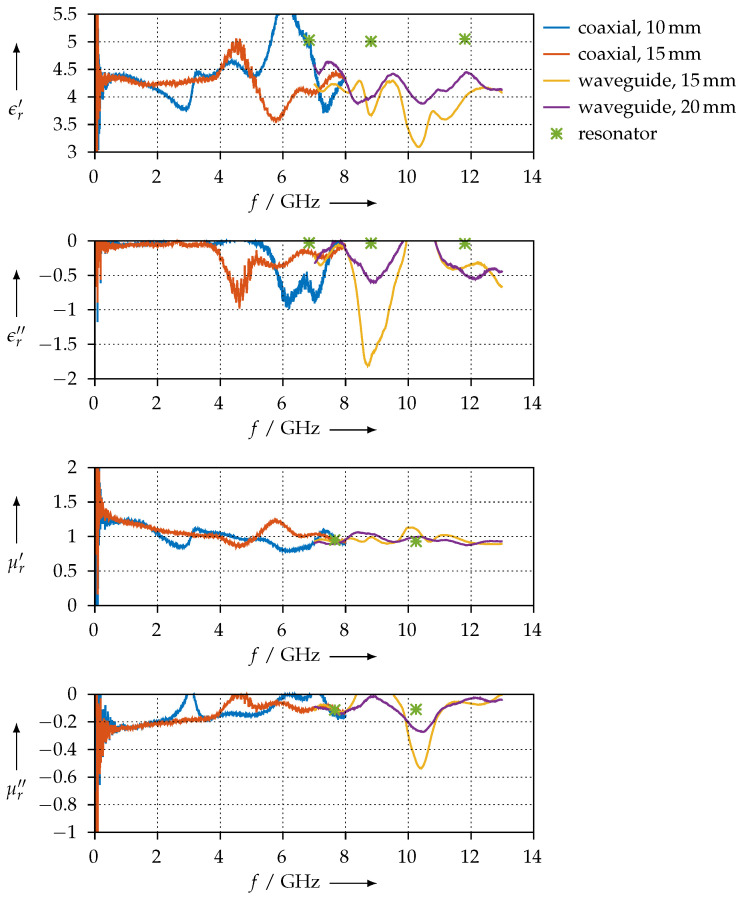
Real ($\epsilon_{r}^{\prime }$, $\mu_{r}^{\prime }$) and imaginary ($\epsilon_{r}^{\prime \prime }$, $\mu_{r}^{\prime \prime }$) parts of the permittivity and permeability for the FEP material.

On the other hand, Figure 22 shows the permittivity and permeability when we use both of them as free parameters. We can observe that, for our frequency range, the magnetic effects seem to vanish, as $\mu_{r}^{\prime }$ is always very close to 1 and $\mu_{r}^{\prime \prime }$ is almost zero. The agreement between the coaxial and the waveguide measurements is very good, but the overall stability of the extraction process is poorer than if we assume ${\hat{\mu }}_{r}=1$.

#### 3.3.3. SSP Material

**Figure 23:**
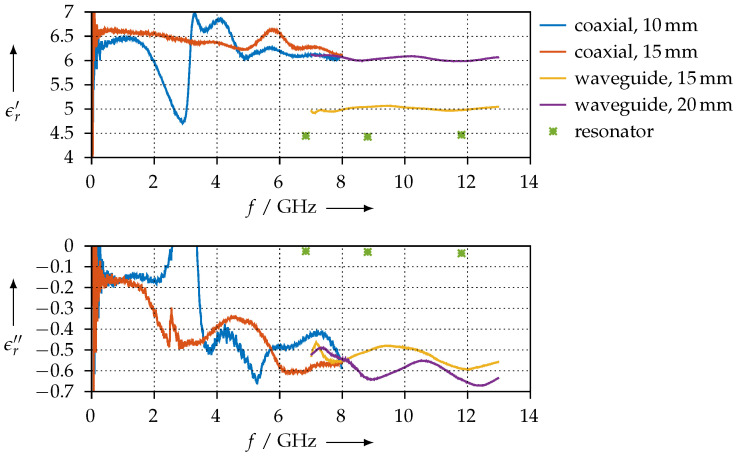
Real ($\epsilon_{r}^{\prime }$) and imaginary ($\epsilon_{r}^{\prime \prime }$) parts of the permittivity for the SSP material.

The permittivity that we extracted for the SSP material is shown in Figure 23. Since this material consists of PLA that is loaded with stainless steel, we do not expect magnetic effects. Nevertheless, we verified this by also extracting both the permittivity and the permeability, with similar results as for the FEP material. Again, we observe a very good agreement between the coaxial and waveguide measurements. However, we also have a large ripple on the coaxial measurements, due to the same reason as for the FEP sample (Section 3.3.2). For the 15-mm waveguide sample, we observe a large deviation of the real part from the other measurements; we do not have an explanation for this, so far as the printing quality of all samples was comparable. One possible explanation could be due to variations of the loading of the PLA matrix with the filler material, stainless steel in this case.

#### 3.3.4. SAAB Material

**Figure 24:**
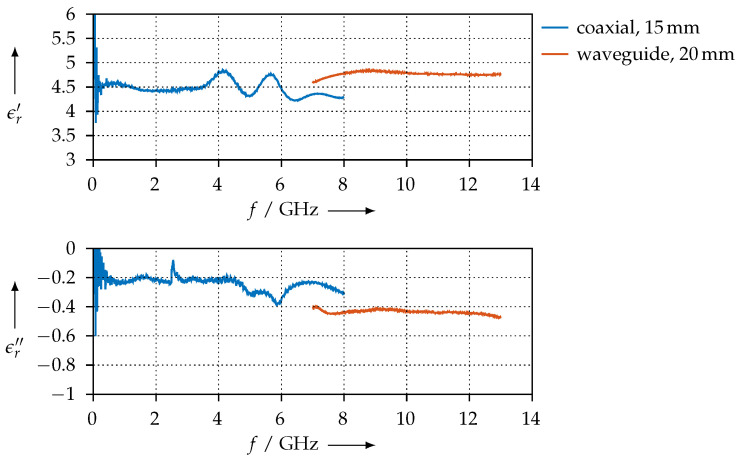
Real ($\epsilon_{r}^{\prime }$) and imaginary ($\epsilon_{r}^{\prime \prime }$) parts of the permittivity for the SAAB material.

Figure 24 shows the permittivity that we have extracted for the SAAB material. In contrast to all other materials we have tested, it seems like this material does not exhibit a strong variation of the permittivity with frequency, i.e., its real and imaginary parts are almost constant from 10 MHz to 13 GHz. Between 7 GHz and 8 GHz, we have less agreement between the coaxial and waveguide measurements than for the other samples we have presented so far. This can be most likely explained by the lower printing quality with this material, as we found it was more difficult to process than the others.

#### 3.3.5. Anisotropic Measurements

**Figure 25:**
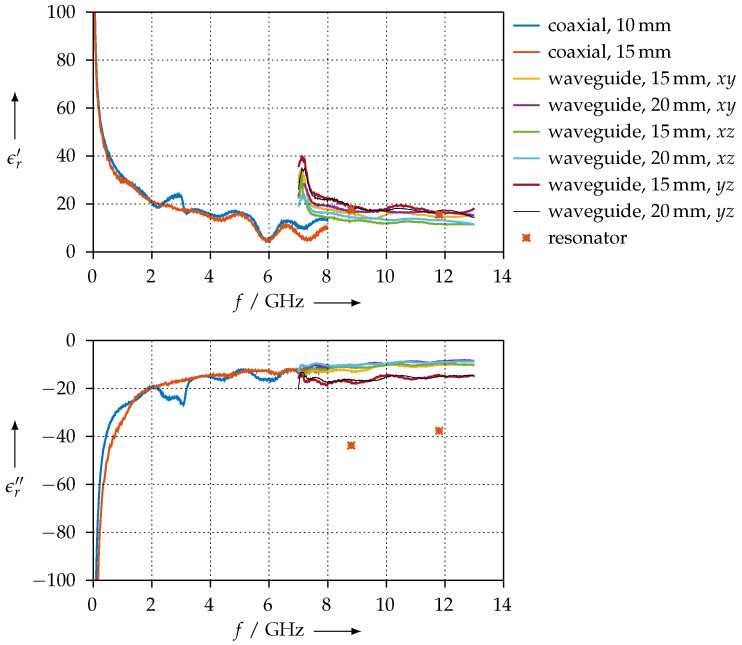
Real ($\epsilon_{r}^{\prime }$) and imaginary ($\epsilon_{r}^{\prime \prime }$) parts of the permittivity for the CDP material for different orientations.

We have measured the anisotropic material parameters for the CDP and KIM materials—these were the easiest to process with our 3D printer and produced the most reproducible results. Figure 25 shows the permittivity for the CDP material for coaxial and all three possible orientations and for different sample lengths.

**Figure 26:**
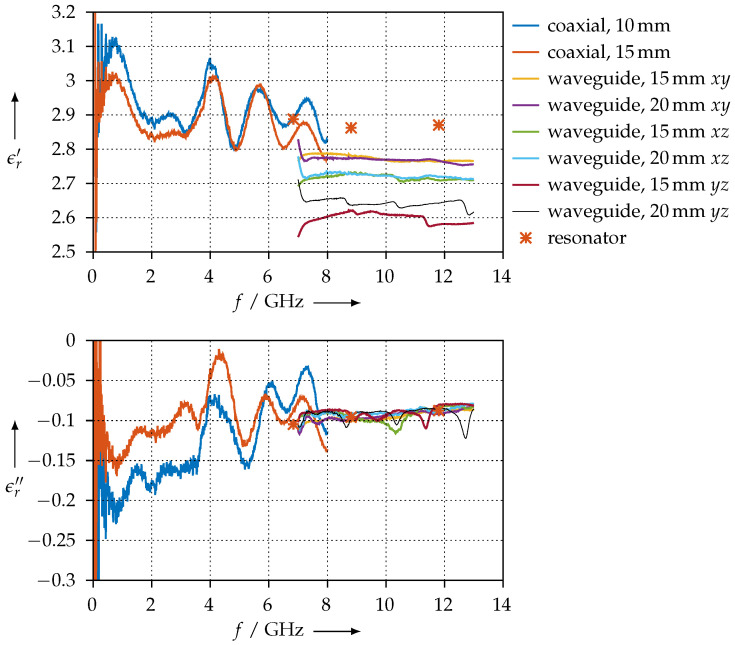
Real ($\epsilon_{r}^{\prime }$) and imaginary ($\epsilon_{r}^{\prime \prime }$) parts of the permittivity for the KIM material for different orientations.

For the KIM material, the permittivity is shown in Figure 26. In contrast to the CDP material, this measurement is much more consistent: we observe that samples having the same orientation exhibit the same permittivity. Furthermore, the real part of the permittivity changes slightly when the printing orientation is altered, but the imaginary part does not show any significant variations. Since the KIM material is a modified ABS, one should compare it with unmodified ABS. We find good agreement in terms of the real part of the permittivity [19,28]. The imaginary part of the KIM material, however, deviates by design from the unmodified ABS, since the modification is intended to increase the DC conductivity and, therefore, also the RF losses.

## 4. Discussion

### 4.1. Measurement Consistency

**Figure 27:**
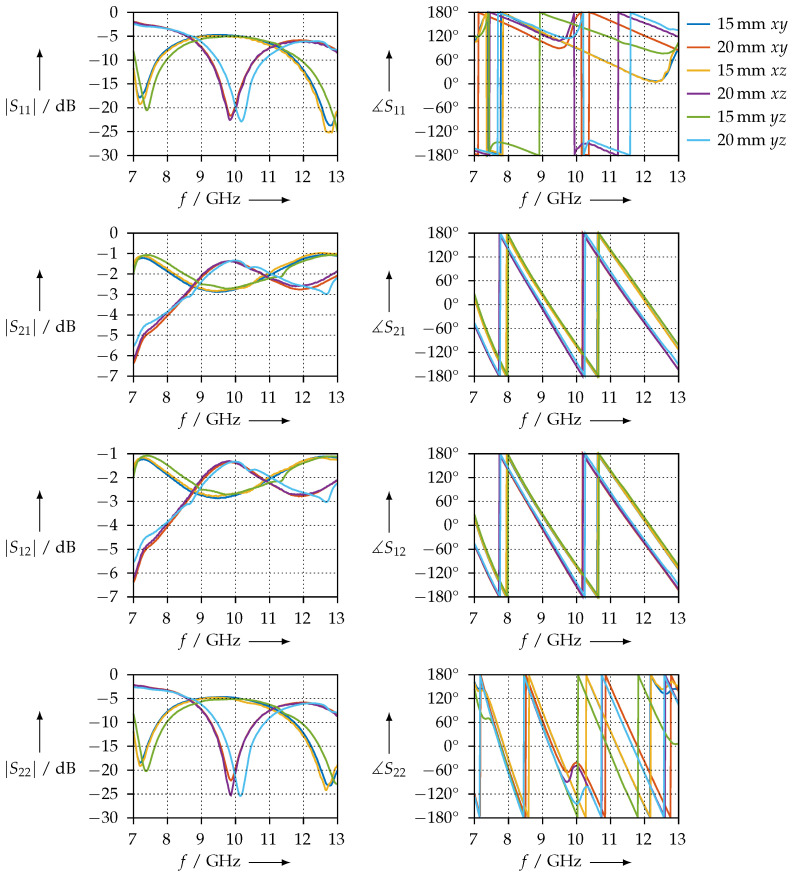
Magnitude and phase of the transmission and reflection S-parameters of the KIM material samples, measured in the WR90 waveguide.

Figure 27 shows the transmission and reflection S-parameters of the KIM material samples in the WR90 waveguide measurement setup. Qualitatively, we observe that $\left|S_{11}\right|\;=\;\left|S_{22}\right|$. This indicates that the material samples are homogeneous such that the reflection coefficients at their faces are equal. We can further observe that $S_{21}=S_{12}$ for both the magnitude and the phase. This is a further indicator that these samples are homogeneous. Otherwise, the phase slopes of $S_{21}$ or $S_{12}$ are almost identical between all samples, from which we can conclude that the real parts of the permittivity are almost equal, which is also observed in our results.

**Figure 28:**
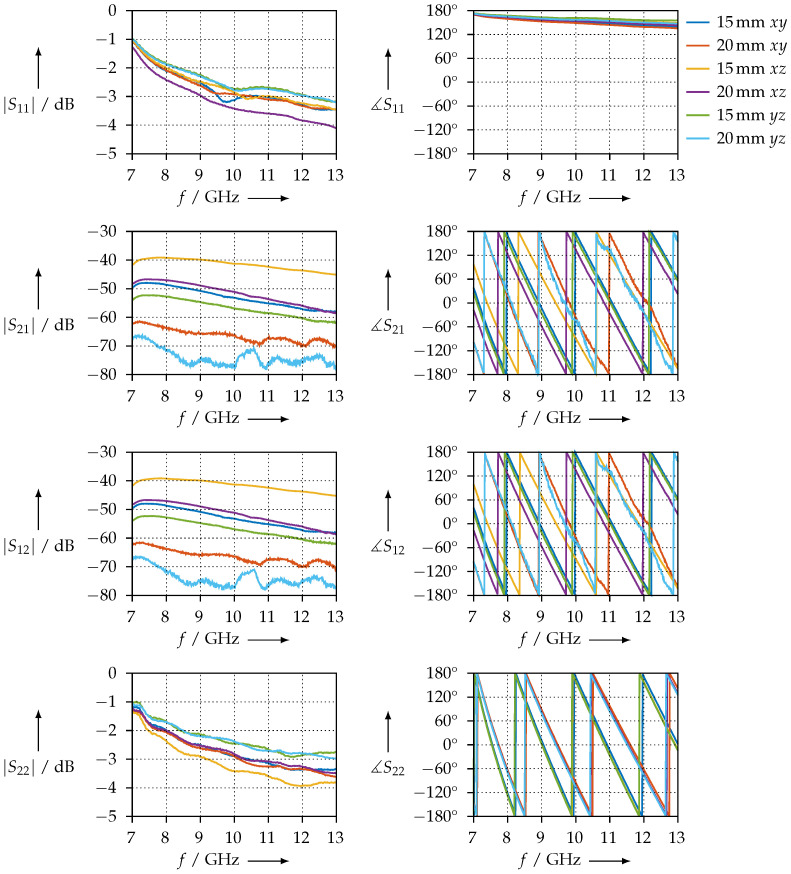
Magnitude and phase of the transmission and reflection S-parameters of the CDP material samples, measured in the WR90 waveguide.

In contrast, Figure 28 shows the same measurements for the CDP samples. We observe that the condition $\left|S_{11}\right|\;=\;\left|S_{22}\right|$ is not met for all samples; this is an indicator that the printing process was not homogeneous. Reasons for this could be an unbalanced distribution of the carbon powder in the filament or irregularities resulting from 3D printing. However, the condition $S_{21}=S_{12}$ is met. The phase slopes of $S_{21}$ or $S_{12}$ are almost identical, which leads us to the assumption that the real parts of the permittivity should be nearly identical as well. To verify whether the inconsistent $\left|S_{11}\right|$ and $\left|S_{22}\right|$ are not measurement artefacts but indeed a property of the sample being measured, we exchanged the waveguide ports, i.e., connected port 2 where port 1 was, and vice versa. We found that we obtained the same results, but with $S_{11}$ and $S_{22}$ (and, of course, $S_{21}$ and $S_{12}$) interchanged; therefore, we can conclude that this is actually a property of the sample.

### 4.2. Agreement of the Methods and Measurement Error Estimation

For material samples that exhibit comparatively low losses, such as the PTFE or KIM samples (see Figure 8 and Figure 26), we observe quite good agreement between the permittivity values that were measured with the resonator method and the one that was measured with the transmission/reflection method. As we explained, the permittivity calculation from resonator measurements is based on the cavity perturbation theory, with the assumption of the perturbation being very small. This assumption is certainly true for PTFE, as this material is known to have very low dielectric losses, and obviously, due to our measurements having good agreement, is also true for the KIM material samples. When the dielectric losses of the MUT are small, the resonator is only slightly perturbed and the calculations derived from the perturbation calculation yield a useful result.

From the PTFE sample, we see that the difference between the permittivity measured with the resonator vs. that measured with the transmission/reflection method is smaller than 0.1. We can use this as an estimate of our measurement error, which then amounts to around 5%. For the 3D-printed materials, the measurement error will be limited by the precision of the printed samples, i.e., due to air pockets, dimension tolerances, and so on.

For materials having high losses, such as CDP (refer to Figure 25), SSP (refer to Figure 23), or FEP (Figure 21 and Figure 22, respectively), the perturbation of the resonator is large enough such that the assumptions of the perturbation method are no longer valid, even though we used small material samples only. This is the reason why we observe only poor agreement of the permittivity measured with the resonator vs. measured with the transmission/reflection method for these materials.

Even though the resonator proved to be unreliable for the measurement of these absorbing materials, it is still a useful method since the resonator can be used to reliably determine whether a material exhibits magnetic properties.

## 5. Conclusions

We have analysed samples of different microwave-absorbing materials which are suitable for 3D printing using the FFF method. SEM images were made so we could observe the constituents of the materials.

Using different measurement techniques based on S-parameter measurements, we extracted the permittivity and permeability of the material samples. Generally, we observed a good agreement between the transmission/reflection method in a waveguide or in a coaxial transmission line setup. The resonator method with cavity perturbation calculations turned out to be unsuitable for these kinds of material, as the perturbation is far too large and, therefore, the results are doubtful compared to the results obtained with the other two methods. Only for material samples having low losses did the resonator provide reliable measurements.

We observed that the real part of the permittivity is more strongly affected by the printing orientation, with respect to the wave propagation, than the imaginary part. However, the samples made with CDP in yz orientation are an exception to this.

Therefore, if 3D-printed materials are to be used for microwave absorbers where a certain specification must be met, care must be taken to use the correct printing orientation of the absorbers.

## Figures and Tables

**Table 1:** Materials that we have examined in this work.

Abbreviation	Material	Filler	Filler wt%	Matrix
PLA	Flashforge PLA standard filament	none	none	PLA
CDP	Protopasta CDP12805	carbon black	<35	PLA
FEP	Protopasta FEP12805	iron	<45	PLA
SSP	Protopasta SSP12805	stainless steel	>60	PLA
SAAB	SAAB Barracuda RAM filament	N/A	N/A	N/A
KIM	Kimya ABS-ESD Black 3D filament	none	none	ABS

**Table 2:** Resonant frequency and *Q* for different modes and samples in the rectangular waveguide resonator and material parameters extracted with Equations (3)–(6).

Sample	Mode	$f_{0}$ or $f_{m}$ (GHz)	$Q_{0}$ or $Q_{m}$	$\epsilon_{r}^{\prime }$	$\epsilon_{r}^{\prime \prime }$	$\mu_{r}^{\prime }$	$\mu_{r}^{\prime \prime }$
empty	TE${}_{101}$	6.851277	2753				
empty	TE${}_{102}$	7.653260	2052				
empty	TE${}_{103}$	8.827069	1492				
empty	TE${}_{104}$	10.245652	1101				
empty	TE${}_{105}$	11.82682	1286				
PTFE	TE${}_{101}$	6.827127	2749	2.0245	−0.0001		
PTFE	TE${}_{102}$	7.653156	2048			1.0070	−0.0003
PTFE	TE${}_{103}$	8.796516	1487	2.0059	−0.0003		
PTFE	TE${}_{104}$	10.245335	1101			1.0070	0
PTFE	TE${}_{105}$	11.785624	1280	2.0125	−0.0006		

**Table 3:** Material samples that we have prepared.

Material	Type	Orientation	Length (mm)	Diameter (mm)
PLA	waveguide	xy	15	
PLA	waveguide	xy	20	
PLA	coaxial	—	10	
PLA	coaxial	—	20	
PLA	resonator	—		0.6
CDP	waveguide	xy	15	
CDP	waveguide	xy	20	
CDP	waveguide	xz	15	
CDP	waveguide	xz	20	
CDP	waveguide	yz	15	
CDP	waveguide	yz	20	
CDP	coaxial	—	10	
CDP	coaxial	—	15	
CDP	resonator	—	30	0.4
FEP	waveguide	xy	15	
FEP	waveguide	xy	20	
FEP	coaxial	—	10	
FEP	coaxial	—	15	
FEP	resonator	—	30	0.8
SSP	waveguide	xy	15	
SSP	waveguide	xy	20	
SSP	coaxial	—	10	
SSP	coaxial	—	15	
SSP	resonator	—	30	0.8
SAAB	coaxial	—	15	
SAAB	coaxial	—	20	
KIM	waveguide	xy	15	
KIM	waveguide	xy	20	
KIM	waveguide	xz	15	
KIM	waveguide	xz	20	
KIM	waveguide	yz	15	
KIM	waveguide	yz	20	
KIM	coaxial	—	10	
KIM	coaxial	—	15	
KIM	resonator	—	30	1.1

**Table 4:** Extracted material parameters for the material samples.

Sample	Mode	$\epsilon_{r}^{\prime }$	$\epsilon_{r}^{\prime \prime }$	$\mu_{r}^{\prime }$	$\mu_{r}^{\prime \prime }$
PLA	TE${}_{101}$	2.76	−0.02		
PLA	TE${}_{102}$			1.01	0
PLA	TE${}_{103}$	2.77	−0.02		
PLA	TE${}_{104}$			1.01	0
PLA	TE${}_{105}$	2.75	−0.02		
CDP	TE${}_{101}$	—	—		
CDP	TE${}_{102}$			1.09	−0.02
CDP	TE${}_{103}$	17.63	−43.76		
CDP	TE${}_{104}$			1.08	−0.01
CDP	TE${}_{105}$	15.52	−37.62		
FEP	TE${}_{101}$	5.03	−0.03		
FEP	TE${}_{102}$			0.95	−0.11
FEP	TE${}_{103}$	5.01	−0.03		
FEP	TE${}_{104}$			0.93	−0.11
FEP	TE${}_{105}$	5.05	−0.04		
SSP	TE${}_{101}$	4.45	−0.03		
SSP	TE${}_{102}$			0.96	−0.10
SSP	TE${}_{103}$	4.43	−0.03		
SSP	TE${}_{104}$			0.94	−0.09
SSP	TE${}_{105}$	4.47	−0.03		
KIM	TE${}_{101}$	2.89	−0.10		
KIM	TE${}_{102}$			1.00	0
KIM	TE${}_{103}$	2.86	−0.10		
KIM	TE${}_{104}$			1.00	0
KIM	TE${}_{105}$	2.87	−0.09		

## Data Availability

The data presented in this study are available on request from the corresponding author.

## Abbreviations

The following abbreviations are used in this manuscript:

ABS	acrylnitrile butadiene styrene
DC	direct current
FFF	fused filament fabrication
MDPI	Multidisciplinary Digital Publishing Institute
MUT	material under test
PLA	polylactic acid
PTFE	polytetrafluoroethylene
RFI	radio frequency interference
SEM	scanning electron microscope
TE	transverse electric
TEM	transverse electromagnetic
MSDS	material safety data sheet
